# How-I-do-it: laparoscopic left medial sectionectomy utilizing a cranial approach to the middle hepatic vein and Laennec’s capsule

**DOI:** 10.1007/s00423-021-02282-x

**Published:** 2021-07-31

**Authors:** Shunya Hanzawa, Kazuteru Monden, Masayoshi Hioki, Hiroshi Sadamori, Satoshi Ohno, Norihisa Takakura

**Affiliations:** Department of Gastroenterological Surgery, Fukuyama City Hospital, 5-23-1 Zao, Fukuyama, Hiroshima 721-8511 Japan

**Keywords:** Laparoscopic live resection, Laennec’s capsule, Laparoscopic anatomic liver resection, Hepatic vein

## Abstract

**Background:**

Laparoscopic anatomic liver resection is technically demanding, given the need to safely isolate the Glissonean pedicles and expose the hepatic veins (HVs) on the liver parenchyma cut surface. Laennec’s capsule is observed around the Glissonean pedicles and root of the HVs. However, its existence, particularly on the peripheral side of the HVs, remains controversial. Herein, we describe Laennec’s capsule-related histopathological findings around the HVs and a safe laparoscopic left medial sectionectomy utilizing Laennec’s capsule.

**Methods:**

The extrahepatic Glissonean approach was performed by connecting Gates II and III, in accordance with Sugioka’s Gate theory. Liver parenchymal transection commenced along the demarcation line, which is between the medial and lateral sections, and the G4 was dissected during transection. Subsequently, via the outer-Laennec approach, the middle hepatic vein (MHV) was exposed from the root side in cranial view, while Laennec’s capsule was preserved. Parenchymal transection was completed while connecting the MHV with the demarcation line. We obtained the membrane surrounding the HVs and performed histopathological examinations.

**Results:**

Six patients underwent laparoscopic left medial sectionectomy from February 2012 to November 2020. There were no cases involving complications (Clavien–Dindo classification; grade II or higher), open-surgery conversion, transfusion, or surgery-related death. The histopathological findings showed Laennec’s capsule surrounding both the trunk of the major HVs and the peripheral side of the HVs.

**Conclusions:**

A cranial approach to the major HVs utilizing Laennec’s capsule is a feasible and advantageous procedure for laparoscopic left medial sectionectomy. We propose that Laennec’s capsule surrounds the entire length of the HVs.

**Supplementary Information:**

The online version contains supplementary material available at 10.1007/s00423-021-02282-x.

## Introduction

Laparoscopic liver resection (LLR) offers advantages over open surgery such as smaller wounds, reduced postoperative pain, reduced blood loss, and its unique laparoscopic and magnified views [1, 2]. However, laparoscopic anatomic liver resection (LALR) is technically demanding given the limited angle of the laparoscope and limited capacity to manipulate the surgical instruments [3] while requiring safe isolation of the Glissonean pedicles and exposure of the hepatic veins (HVs) on the cut surface of the liver parenchyma [4–6]. To safely isolate the Glissonean pedicles and expose the HVs, it is essential to understand the anatomy around the Glissonean pedicle and the HVs [7].

**Fig. 1:**
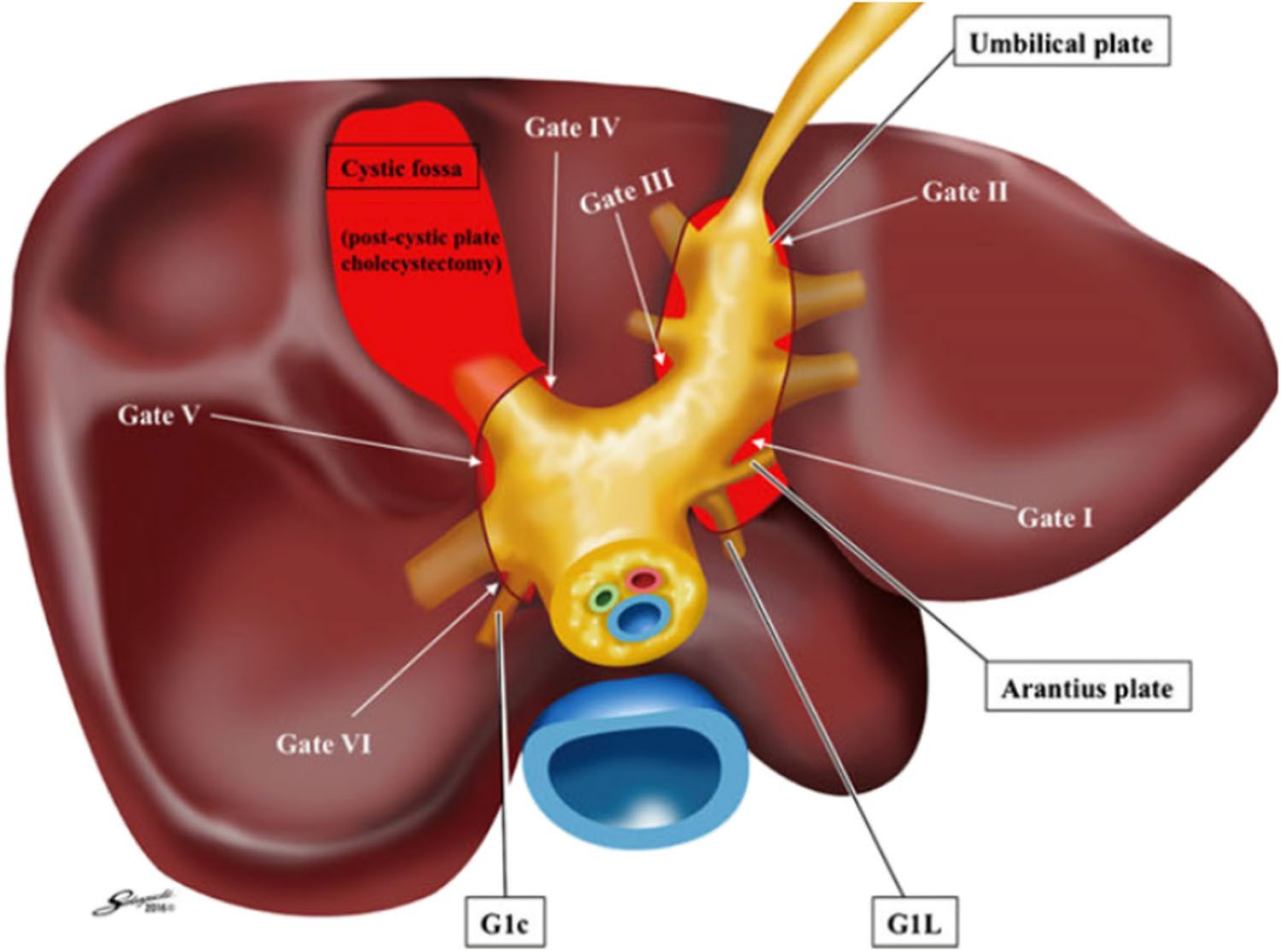
Sugioka’s Gate theory. The schema shows four anatomical landmarks, six gates, and Laennec’s capsule. Gate II represents the junction between the round ligament and the umbilical plate, and Gate III represents the right edge of the Glissonean pedicle root of the umbilical portion. The red area represents Laennec’s capsule. G1c, the Glissonean pedicle of the caudate process; G1L, the Glissonean pedicle of the Spiegel lobe. This figure was adapted from the following report: Sugioka A, Kato Y, Tanahashi Y (2017) Systematic extrahepatic Glissonean pedicle isolation for anatomical liver resection based on Laennec’s capsule: proposal of a novel comprehensive surgical anatomy of the liver. J Hepatobiliary Pancreat Sci 24:17–23. https://doi.org/10.1002/jhbp.410

Laennec’s capsule was first described by R.T.H. Laennec in 1802 as a thin fibrous membrane covering the entire surface of the liver, including the Glissonean pedicles and HVs [8]. Recently, Sugioka et al. [7] introduced systematic extrahepatic Glissonean isolation based on Laennec’s capsule. They showed that by connecting the “Gates” (defined by anatomic landmarks) (Fig. 1), the Glissonean pedicles could be isolated without parenchymal destruction. In locating Laennec’s capsule, several authors [7, 9, 10] have reported histopathological findings of Laennec’s capsule around the HVs. Of these, Hayashi et al. [9] and Monden et al. [10] showed that Laennec’s capsule surrounds not only the trunk of the major HVs but also the peripheral sides of the HVs. However, there are claims, such as those from Shirata et al. [11, 12], that Laennec’s capsule is absent around the peripheral side of the HVs. Although some evidence in the existing literature hint at the presence of Laennec’s capsule on the peripheral side of the HV, its existence remains highly contested among clinicians.

Herein, we describe histopathological findings that hint at the presence of Laennec’s capsule around the HVs (both the common trunk of the major HVs and the peripheral side of the HVs) and a safe laparoscopic left medial sectionectomy utilizing Laennec’s capsule.

## Methods

### Surgical approach

**Fig. 2:**
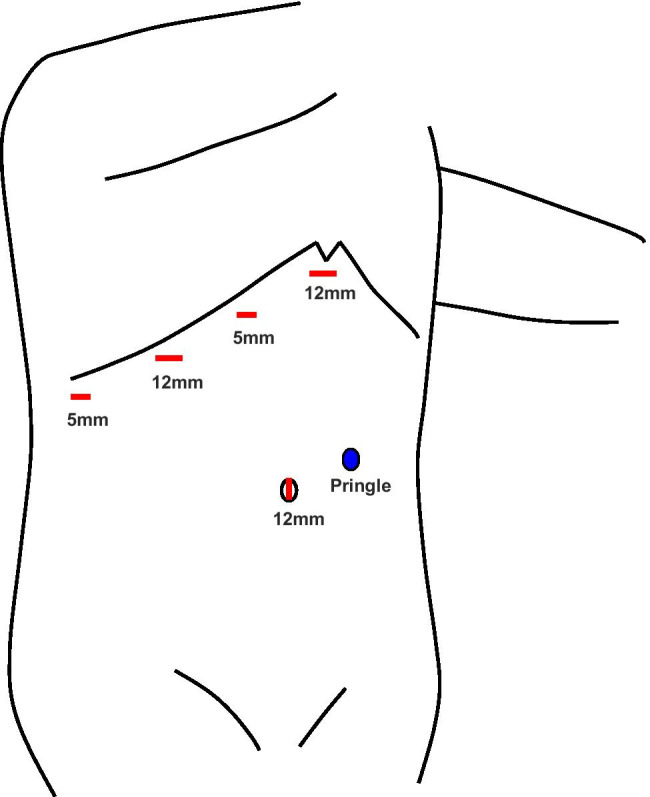
Trocar placement. Two 12-mm trocars and two 5-mm trocars are placed below the right costal arch. A tourniquet for the Pringle maneuver is placed in the left lateral abdomen

Patients were placed in the left hemilateral position. The main surgeon was positioned on the right side of the patient at the beginning of the surgery. The pneumoperitoneum pressure (PPP) was maintained at 10 mmHg. The first trocar (12-mm) for the laparoscope was inserted through the umbilicus. Next, two 12-mm trocars and two 5-mm trocars were placed below the right costal arch. A tourniquet was prepared for the Pringle maneuver through the left lateral part of the abdomen (Fig. 2).

**Fig. 3:**
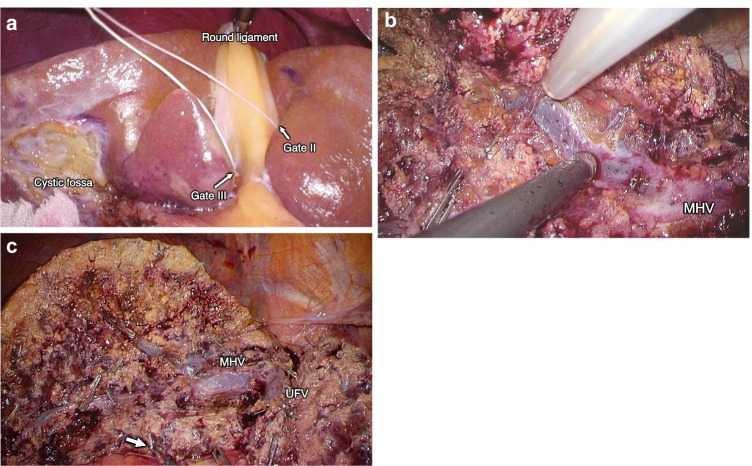
Intraoperative images of laparoscopic left medial sectionectomy. **a** By connecting Gate II and Gate III, the Glissonean pedicle of segment 4 was isolated. **b** The middle hepatic vein was exposed from the root side in the cranial view. **c** The middle hepatic vein was exposed after the left medial sectionectomy. White arrow indicates the stump of the Glissonean pedicle of segment 4. MHV, middle hepatic vein; UFV, umbilical fissure vein

After cholecystectomy, the extrahepatic Glissonean pedicle isolation was performed utilizing Sugioka’s Gate theory [7]. The Glissonean pedicle of segment 4 (G4) was isolated extrahepatically without parenchymal destruction by connecting the left side of the umbilical portion (UP) of Gate II and Gate III (Fig. 3a). When connecting the right side of the UP of Gate II and Gate III, some G4 branching from the cranial side of the UP may remain. In contrast, by connecting the left side of the UP of Gate II and Gate III, most G4s can be included and isolated at once (supplemental video). In addition, it is essential to confirm that Laennec’s capsule is on the liver side as a shiny membrane and not entering the Glissonean sheath.

After isolating the entire G4 and temporally clamping, the demarcation was apparent as a dissection line. The G4 was dissected using a vascular stapler after ensuring that liver dissection progressed to the point where the Glissonean pedicle could be safely and reliably dissected. Thereafter, the main surgeon shifted to the left side of the patient. Liver parenchymal transection was performed along the Rex–Cantlie line, therein exposing the middle hepatic vein (MHV) from the root side in the cranial view (Fig. 3b). The root of the MHV was identified extrahepatically; we could enter the intersectional plane by tracing the MHV from the root. The Cavitron Ultrasonic Surgical Aspirator (CUSA) was subsequently moved from the root side toward the peripheral side of the HV while avoiding the splitting of the bifurcation of the HV. In addition, in the cranial view, it was easier to confirm Laennec’s capsule by retaining it on the IVC and MHV side (the outer-Laennec approach), which served to maintain the strength of the vein’s wall.

After exposing the full length of the MHV, an assistant lifted the resected liver to the right upper space, and the liver parenchyma was subsequently transected while connecting the hepatic vein via the demarcation. The parenchymal transection was completed (Fig. 3c), and the resected liver was removed in a plastic bag through the extended umbilical port incision. Subsequently, a drain was placed on the liver transecting plain.

## Results

Between February 2012 and November 2020, we performed six laparoscopic left medial sectionectomies out of 165 LALRs (Table 1). The median operation time was 371 min (range, 210–444 min), and median blood loss was 150 mL (range, 50–600 mL). The median postoperative length of hospital stay was 7.5 days (range, 6–12 days). There was no need for open conversion, and no major complications occurred (Clavien–Dindo classification; grade II or higher).

**Table 1:** Clinical and surgical characteristics of treated patients

Patient no	Operation procedure	Tumor type	Oncological quality	Operation time, minutes	Amount of blood loss, mL	Length of stay, days	Complications
1	Left medial sectionectomy with resection of the ventral part of S8	CRLM	R0	268	150	8	None
2	Left medial sectionectomy with resection of the MHV	CRLM	R0	404	150	7	None
3	Left medial sectionectomy with resection of the MHV and ventral part of S8	HCC	R0	444	600	7	None
4	Left medial sectionectomy	CRLM	R0	210	50	8	None
5	Left medial sectionectomy	CRLM	R0	339	50	6	None
6	Left medial sectionectomy with resection of the MHV Partial resection of S7 and S8	CRLM	R0	410	400	12	None
Median	-	-	-	371.5	150	7.5	-

*CRLM* colorectal liver metastasis; *HCC* hepatocellular carcinoma; *MHV* middle hepatic vein; *S7* segment 7; *S8* segment 8

**Fig. 4:**
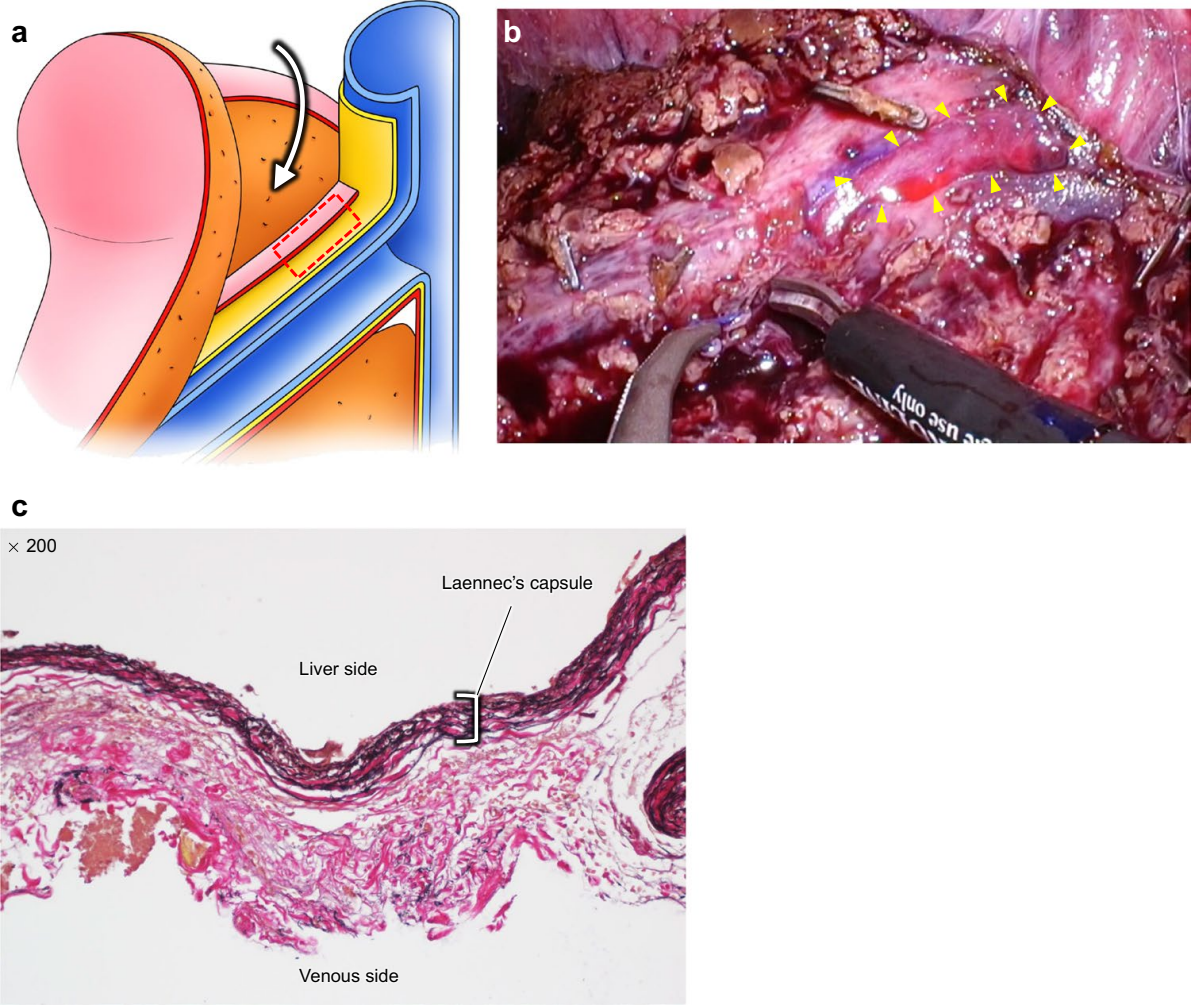
Schema of liver anatomy based on Laennec’s capsule and Laennec’s capsule-related histopathological findings at the trunk of the major hepatic vein. **a** Schema shows the exposure of the hepatic veins (HVs) from the root side to the peripheral side in the outer-Laennec approach. In this approach, Laennec’s capsule is preserved on the HV side. Laennec’s capsule is obtained from the red-dotted area. **b** Intraoperative image showing the exposure of the HVs. A small specimen was obtained from the area indicated by the yellow arrowheads on the intraoperative image. **c** Image showing the pathological findings obtained in Fig. 4b (the area indicated by the yellow arrowheads). Histopathological analysis of the trunk of the HV shows a wavy elastic fiber (blackish purple) and collagen fiber (red) (Elastica Van Gieson staining, 200 × magnified view)

Histopathological findings of Laennec’s capsule dissected from the HV trunk are shown in Fig. 4. We have explained how to expose the HVs from the root side to the peripheral side while preserving Laennec’s capsule using the outer-Laennec approach in the schema (Fig. 4a). Laennec’s capsule was dissected and obtained from the root side of the HV (Fig. 4b). Elastica Van Gieson staining showed that the thick Laennec’s capsule around the wall of the major HV was composed of a mixture of elastic and collagen fibers (Fig. 4c).

**Fig. 5:**
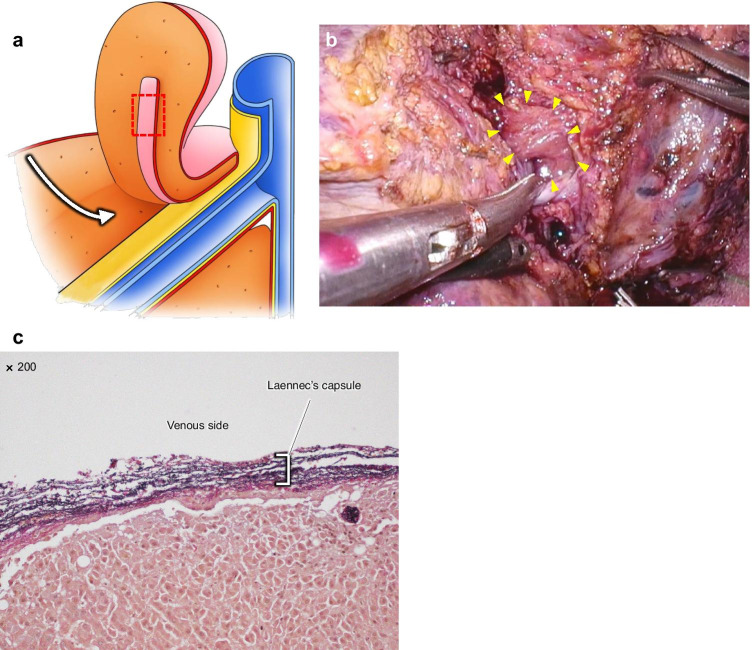
Schemas of the liver anatomy based on Laennec’s capsule and Laennec’s capsule-related histopathological findings at the peripheral side of the major hepatic vein. **a** Schema showing the exposure of the hepatic veins (HVs) from the peripheral side to the root side in the caudal approach. Laennec’s capsule is observed to be peeling off from the HV. Laennec’s capsule is obtained from the red-dotted area. **b** Intraoperative image showing Laennec’s capsule, which is peeled off from the HV. A small specimen was obtained from the area indicated by the yellow arrowheads on the intraoperative image. **c** Image showing the pathological findings obtained in Fig. 5b (the area indicated by the yellow arrowheads). Histopathological analysis of the peripheral side of the HV shows a wavy elastic fiber (blackish purple) and collagen fiber (red) on the liver side (Elastica Van Gieson staining, 200 × magnified view)

In contrast, Laennec’s capsule is thinner on the peripheral side of the HVs. Laennec’s capsule tends to easily peel off the HVs when the hepatic vein is exposed from the peripheral side to the root side (Fig. 5a), resulting in HV fragility. In another case, Laennec’s capsule was accidentally peeled off along the HV from the peripheral side to the root side, resulting in Laennec’s capsule being attached to the liver side (i.e., a liver specimen with Laennec’s capsule attached). Histopathological findings, in this case, revealed elastic and collagen fibers attached to the parenchymal side of the liver (Fig. 5b, c).

## Discussion

In the current study, we described a safe procedure for isolating the Glissonean pedicles and exposing the MHV from the root side by utilizing Laennec’s capsule in laparoscopic left medial sectionectomy. To the best of our knowledge, no previous reports have described laparoscopic left medial sectionectomy performed with the extrahepatic Glissonean approach based on Sugioka’s Gate theory. We further described the histopathological findings of Laennec’s capsule around the HVs.

Anatomic liver resection is preferable and has better oncological curability in patients with hepatocellular carcinoma, as metastasis generally occurs through the portal venous system [13, 14]. For metastatic liver tumors, we mainly perform parenchymal-sparing hepatectomy; however, for cases in which the tumor is adjacent to the Glissonean pedicles or the patient has portal vein thrombosis, we perform anatomic liver resection. Previous studies have suggested that LALR is beneficial in treating metastatic liver tumors located deep within the tissue. In such cases, the surgical margins can be secured by identifying the HVs or Glissonean pedicles adjacent to the tumor and exposing them to the cutting plane as landmarks, thereby improving safety and efficacy [2, 4].

LALR, including left medial sectionectomy, is still considered a challenging procedure for the following two reasons. The first concerns the isolation of the Glissonean pedicle, which will be conducive toward identifying the target segments or sections. The second concerns the careful exposure of the HVs on the cutting surface of the liver [4–6]. These procedures have to be performed within the limited operative field, limited angle of the laparoscope, and limited capacity to manipulate the surgical instruments [3].

Our approach to laparoscopic left medial sectionectomy mainly included two procedures: extrahepatic Glissonean pedicle isolation and exposure of the HVs from the root side with a cranial view. Our approach to extrahepatic Glissonean pedicle isolation was informed by Sugioka et al. [7] who proposed a novel and comprehensive anatomy of the liver based on Laennec’s capsule. This theory has significantly contributed toward standardizing the operative procedures, including through the development of the Gate theory. The Gate theory enabled us to isolate the extrahepatic Glissonean pedicle without damaging the liver parenchyma by leveraging the anatomical gap between the Glissonean pedicle and Laennec’s capsule. This concept can enable safe isolation and dissection of the G4 in laparoscopic left medial sectionectomy.

Laennec’s capsule covers the Glissonean pedicles and surrounds the HVs, as first described by R.T.H. Laennec [8]. Monden et al. [10, 15] and Kiguchi et al. [16, 17] further introduced the outer-Laennec approach. This approach can safely expose the HVs, serving to maintain the strength of the vein wall because Laennec’s capsule is preserved on the HV side. Our histopathological findings suggest that Laennec’s capsule surrounds the entire length of the HVs on the peripheral side. These findings have important therapeutic implications. If the tumor is close to the HVs, attaching Laennec’s capsule to the side of the tumor can secure the surgical margin by retaining Laennec’s capsule on the excision side of the liver parenchyma (i.e., the tumor side) [10, 15].

We performed laparoscopic left medial sectionectomy utilizing this approach in six cases, and no major complications or mortality was observed. However, our study had some limitations. First, the operation required a long time as most patients underwent extended left medial sectionectomy, such as combined resection of the MHV, ventral part of S8, or additional partial resections. Second, the length of hospital stays tended to be long because of the particularities of the medical insurance system in Japan. Finally, although our study did not include a large sample size, we provided evidence supporting the safety and usefulness of the presented procedure and illustrated its potential for standardization.

Although transection from the root of the HVs poses potential bleeding risks [18], these can be minimized by utilizing Laennec’s capsule and some technical tips. First, large injuries in the root of the HVs require suture closure, while minor injuries, such as small holes, can be controlled with soft coagulation [19]. This is because Laennec’s capsule around the trunk of the major HVs is thick and denatured with soft coagulation, resulting in the closure of the bleeding point [10]. Second, split injury, caused by the bifurcation of the HVs, is the most critical injury [2]. Therefore, the CUSA would be better moved from the root to the periphery [20]. Split injury can be avoided by conducting parenchymal liver dissection via the root side of the HVs.

Many reports have been recently published with an aim to standardize LALR procedures based on the concept of Laennec’s capsule [10, 16, 17, 21–23]. In addition to these previous surgical techniques, our left medial sectionectomy approach also has potential for use in other LALRs and as a safe and curative method to prevent intraoperative complications, such as unexpected massive bleeding. Moreover, our work provides additional histopathological evidence of Laennec’s capsule, which will contribute toward the use of Laennec’s capsule for the standardization of surgical procedures and the development of novel surgical techniques. We believe that LALRs based on Laennec’s theory will become widespread in the near future. However, larger investigations focusing on histological findings are still needed to improve its safety.

## Conclusion

We described a cranial approach for exposing the major HVs based on the concept of Laennec’s capsule and demonstrated its safety and feasibility for laparoscopic left medial sectionectomy. In addition, our histopathological findings suggest that Laennec’s capsule surrounds the hepatic veins from the root to the peripheral side.

## Supplementary Information

Below is the link to the electronic supplementary material.

Supplementary file1 (MP4 244232 KB)

## Data Availability

Data is available on request from the corresponding author.
